# Standardization of Immunohistochemistry Using an Anti‐WASLB Antibody in the Skeletal Muscle of Teleost Fish

**DOI:** 10.1002/cpz1.70404

**Published:** 2026-06-16

**Authors:** Erika Stefani Perez, Matheus Naia Fioretto, Bruna Tereza Thomazini Zanella, Rafael Henrique Nóbrega, Bruno Oliveira da Silva Duran, Maeli Dal Pai‐Silva

**Affiliations:** ^1^ São Paulo State University (UNESP) Botucatu Brazil; ^2^ Federal University of Jataí Goiás Brazil; ^3^ Federal University of Goiás Goiânia Brazil

**Keywords:** fish skeletal muscle, immunohistochemistry, pacu, specific antibody, zebrafish

## Abstract

Immunohistochemistry (IHC) is a widely used technique for the identification, localization, and analysis of protein expression in tissues, playing a critical role in pathological diagnosis and validation of data derived from molecular biology. Despite its well‐established use in mammalian tissues, applying IHC to fish still presents challenges, particularly due to the use of heterologous antibodies and the presence of paralogous genes. In this context, the present study aimed to standardize an immunohistochemistry protocol adapted for fish skeletal muscle using a biotinylated monoclonal antibody designed against a conserved region of the *waslb* gene for pacu (*Piaractus mesopotamicus*) and zebrafish (*Danio rerio*). Muscle samples were fixed in 4% paraformaldehyde, embedded in paraffin, and subjected to heat‐induced antigen retrieval. After blocking nonspecific binding sites, histological sections were incubated with the primary antibody at an optimized 1:50 dilution, followed by detection with fluorophore‐conjugated streptavidin (1:200) and nuclear counterstaining with DAPI. Reaction specificity was assessed using negative controls with a 20,000‐fold molar excess of the immunizing peptide. The standardized protocol resulted in specific and reproducible labeling of the *waslb* protein in the skeletal muscle of both species, with no signal observed in negative controls; however, it is important to emphasize that this protocol can also be applied to antibodies against other fish genes. Thus, this method represents a reliable tool for protein analysis in fish muscle tissues, contributing to physiological, experimental, and comparative studies in non‐mammalian models. © 2026 The Author(s). *Current Protocols* published by Wiley Periodicals LLC.

**Basic Protocol**: Immunohistochemical reaction against the WASLB antibody in paraffin‐embedded skeletal muscle sections of pacus and zebrafish

## INTRODUCTION

Immunohistochemistry (IHC) is a well‐established technique based on the specific interaction between antibodies and antigens, enabling the localization and qualitative or semi‐quantitative analysis of protein expression within cells and tissues (Taylor, 2014). In both biomedical and basic research, IHC remains a gold‐standard approach to validate findings derived from imaging, molecular biology, and transcriptomic analyses. Despite its broad applicability, successful IHC critically depends on antibody specificity, tissue processing, and appropriate control strategies (Mebratie & Dagnaw, 2024).

In skeletal muscle research, IHC is widely used to identify protein alterations associated with myopathies, muscle remodeling, and fiber‐type composition (Danielsson & Häggqvist, 2021; Murach et al., 2019; Suriyonplengsaeng et al., 2017). While standardized IHC protocols and validated antibodies are readily available for mammalian models (Kosmac et al., 2018; Thomas et al., 2025), their direct application to non‐mammalian vertebrates, particularly teleost fish, remains challenging. Teleost genomes have undergone additional rounds of whole‐genome duplication, resulting in a high prevalence of paralogous genes (Garcia de la Serrana et al., 2014). Consequently, antibodies developed against mammalian antigens are frequently heterologous when applied to fish tissues, often leading to cross‐reactivity and nonspecific labeling.

To overcome these limitations, the validation of specific antibodies, combined with immunohistochemical controls, is essential. However, detailed, reproducible IHC protocols tailored to fish skeletal muscle remain scarce in the literature. This methodological gap limits protein‐level analyses in non‐model fish, such as pacu (*Piaractus mesopotamicus*), despite their growing importance in skeletal muscle research, aquaculture, comparative physiology, and translational research.

Compared with western blotting and transcriptomic approaches, immunohistochemistry preserves tissue architecture and enables spatial localization of proteins at the cellular and subcellular levels (Taylor et al., 2010; Taylor, 2014). Although it does not provide absolute quantification, fluorescence‐based detection combined with image analysis allows semi‐quantitative assessment of protein distribution and relative abundance across muscle fiber types. However, the technique is highly dependent on antibody specificity and optimized fixation conditions, which can limit reproducibility when heterologous antibodies are used (Uhlén et al., 2016).

In general, IHC begins with antigen retrieval. This step is crucial to ensure the sensitivity and effectiveness of the technique, as it allows antigens to be accessible to antibody binding. Both physical and chemical methods are used to break the cross‐links formed between the tissue and the fixative. Among physical methods, heat and ultrasound are the most used and may be combined with chemical digestion using enzymes and denaturing agents (Shi et al., 1991).

The next step involves the addition of the primary antibody to the tissue, which may be monoclonal or polyclonal. Monoclonal antibodies are characterized by binding to a single antigen, whereas polyclonal antibodies are less specific and can bind to more than one antigen (Coons et al., 1941; Lin & Chen, 2014; Taylor, 2014). The dilution of primary antibodies varies by antibody type and is generally recommended by the manufacturer. However, each researcher typically determines the optimal antibody dilution based on the tissue analyzed, which is often reported in scientific articles.

To observe the binding between the primary antibody and the antigen, either the primary antibody itself or a secondary antibody that binds to the primary antibody must be labeled, corresponding to the direct and indirect methods, respectively. In the direct method, the primary antibody is synthesized with a label. In the indirect method, the primary antibody is unlabeled, while the secondary antibody carries the label and can be used to bind multiple primary antibodies. This labeling consists of enzymes that produce a color reaction upon addition of a substrate or fluorescent molecules that are visualized under a fluorescence microscope (Taylor et al., 2010; Taylor, 2014).

Finally, to assess the quality and effectiveness of the IHC reaction, a negative control is performed by omitting the primary antibody or by incubating the tissue with a solution containing the antibody and the immunizing peptide (Taylor, 2014). The concentrations used in this solution vary according to the chemical characteristics of the antibody and the peptide.

Here, we report the optimization and validation of an immunohistochemical protocol for teleost skeletal muscle, using pacu and zebrafish (*Danio rerio*) as models. Detection was performed using a biotinylated monoclonal antibody targeting a conserved epitope of the *waslb* gene product. By combining optimized antigen retrieval, fluorescence‐based detection, and a stringent peptide‐blocked negative control, this protocol provides a reliable framework for protein localization in teleost skeletal muscle and can be readily adapted to other fish species and molecular targets.

## STRATEGIC PLANNING

**Figure 1:**
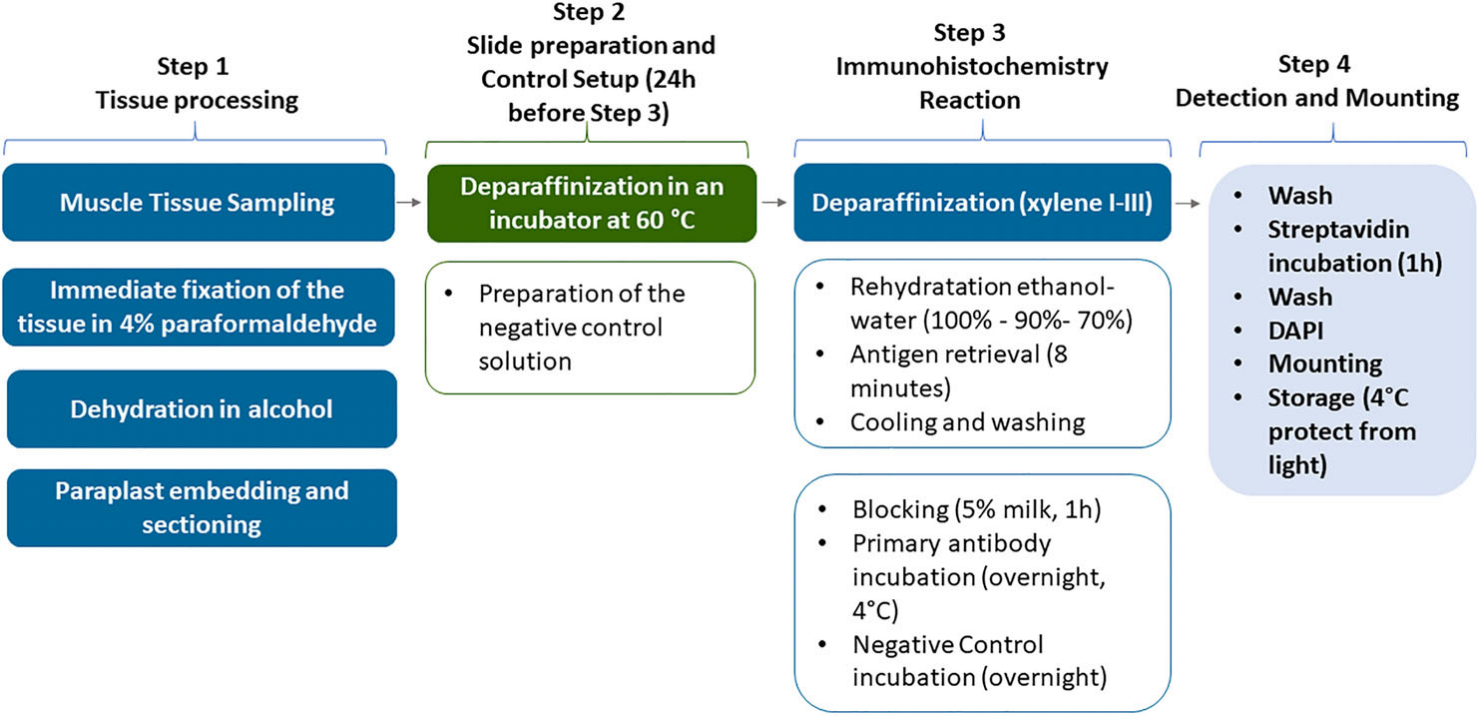
Overview of the Basic Protocol.

See Figure 1 for an overview of the Basic Protocol.

*CAUTION*: The steps of the protocol involving paraformaldehyde, xylene, and alcohol must be carried out in a chemical fume hood (laboratory exhaust hood commonly used in histology laboratories), with the appropriate use of personal protective equipment (PPE), such as masks, nitrile gloves, and a lab coat.

*CAUTION*: It is essential that there be no light exposure during the periods in which the streptavidin being used.

*NOTE*: All protocols involving animals must be reviewed and approved by the appropriate Animal Care and Use Committee and must follow regulations for the care and use of laboratory animals.

## IMMUNOHISTOCHEMICAL REACTION AGAINST THE WASLB ANTIBODY IN PARAFFIN‐EMBEDDED SKELETAL MUSCLE SECTIONS OF PACUS AND ZEBRAFISH

This protocol describes, in detail, the steps required to perform an immunohistochemical reaction on paraffin‐embedded histological sections of skeletal muscle from pacu and zebrafish, using an antibody synthesized from the transcript sequence of the target gene. The antibody was produced conjugated to biotin in order to amplify the signal and eliminate the need for a secondary antibody. If conducted properly, researchers should obtain a clear and specific IHC reaction using straightforward steps. It is important to emphasize that knowing the sequence of the chosen target is essential.

### Materials

Epaxial white muscle samples from pacu and zebrafish
4% paraformaldehyde (PFA) in phosphate‐buffered saline (PBS) or distilled water, freshly prepared or commercial solution, store at 4°C (purpose: fixative; preserves tissue morphology via protein cross‐linking)
1× PBS pH 7.2 to 7.4 (Current Protocols, 2006) (store at room temperature or 4°C; purpose: buffer preparation and washing)
H_2_O, distilled (ready to use; store at room temperature; purpose: dilutions and rinsing)
Ethanol:
  70% ethanol (70 ml of 100% ethanol + 30 ml distilled water; store at room temperature; purpose: tissue storage after fixation, graded dehydration)
  100% commercial reagent (store at room temperature; purpose: removes residual xylene, initiates rehydration)
  90% ethanol (90 ml of 100% ethanol + 10 ml distilled water; store at room temperature; purpose: intermediate graded rehydration)
  70% ethanol (rehydration) (70 ml of 100% ethanol + 30 ml distilled water; store at room temperature; purpose: continues progressive rehydration)
Paraplast (Sigma‐Aldrich, cat. no. P3558), commercial paraffin embedding medium (store at room temperature; purpose: tissue embedding)
Negative control solution [primary antibody + bovine serum albumin (BSA) + 20,000 × peptide excess; incubate 24 hr; store at 4°C; purpose: confirms specificity]
Primary antibody, anti‐WASLB (Rhea Biotech, cat. no. IM‐1049) (store stock at −20°C; dilute 1:50 in BSA; purpose: specific antigen detection)
BSA (Sigma‐Aldrich, cat. no. A7906) (prepare per manufacturer; store at room temperature or 4°C; purpose: antibody stabilization)
Xylene (Sigma‐Aldrich, cat. no. 534056), histological grade (store at room temperature in chemical cabinet; purpose: deparaffinization, dissolves paraffin)
Xylene/ethanol (1:1, mix equal volumes; store at room temperature; purpose: solvent transition step)
50× EnVision FLEX target retrieval solution (DAKO, cat. no. K8004) (store stock at room temperature; dilute 4 ml in 196 ml distilled water for a 1× working solution; purpose: alkaline buffer for HIER)
20× EnVision FLEX wash buffer (DAKO, cat. no. K8000) (store stock at room temperature; dilute 1:20 before use; purpose: removes excess reagents, pH normalization)
5% powdered milk blocking solution (10 g milk in 200 ml PBS; store at 4°C for short term; purpose: blocks nonspecific binding sites, reduces background staining)
Streptavidin–Alexa Fluor 405 (BioLegend, cat. no. 405240) (0.5 mg/ml; prepare per manufacturer; store at 4°C protected from light; purpose: fluorescent detection)
DAPI (Vector Laboratories, cat. no. H1200) (prepare per manufacturer; store at 4°C protected from light; purpose: nuclear counterstain)
Mounting medium (fluorescence‐compatible; ready to use; store at room temperature or 4°C; purpose: preserves fluorescence)
Colorless nail polish (ready to use; store at room temperature; purpose: seals coverslips)

10‐ to 1000‐µl adjustable volume micropipettes (purpose: accurate reagent application)
Freezer, −20°C (purpose: antibody stock storage)
Plastic sample containers (small containers for fixation/ethanol storage; purpose: tissue handling)
Rotary microtome (Leica, cat. no. RM2235 or equivalent) (purpose: cutting 5‐µm paraffin sections)
Silanized glass slides (commercial pre‐treated slides; purpose: improves tissue adhesion)
Incubator, 60°C (purpose: promotes slide adhesion before deparaffinization)
Heat‐resistant staining container compatible with pressure heating (purpose: contains retrieval buffer and slides)
Pressure cooker (Electrolux, cat. no. PCE20) [laboratory‐compatible heat device; purpose: heat‐induced epitope retrieval (HIER)]
Laboratory shaker (orbital or platform shaker; purpose: dissolving blocking solution)
Vortex mixer (standard laboratory vortex; purpose: homogenizing antibody solution)
Refrigerator, 4°C (purpose: storage of antibodies and slides)
Humidified dark box (light‐protected chamber with moist paper; purpose: overnight antibody incubation)
Light‐protected slide storage (aluminum foil or dark slide box; purpose: prevents photobleaching)
Coverslips (standard microscopy coverslips; purpose: section mounting)
Fluorescence microscope (Leica, cat. no. LAS AF 6000)

#### Tissue collection, fixation, dehydration, clearing, and paraffin embedding

1 Collect epaxial white muscle samples from pacu and zebrafish and immediately fix them in 4% paraformaldehyde for 24 hr (other regions of the muscle tissue may also be collected and used).
2 Transfer the fixed samples to 70% ethanol in small plastic containers.
3 Embed the samples in Paraplast.
4 Cut 5‐µm‐thick sections using a rotary microtome.
5 Mount the sections on silanized glass slides.
6 Prepare the negative control solution by mixing the primary antibody and BSA with a 20,000‐fold molar excess of immunizing peptide relative to the antibody (24 hr before step 30).
  The excess peptide saturates the antigen‐binding sites of the antibody, preventing binding to tissue epitopes and confirming staining specificity.
7 Keep the slides containing the histological sections in an incubator at 60°C for 24 hr before deparaffinization to promote proper section adhesion to the slides and facilitate complete paraffin removal.
8 Transfer the slides to xylene II for 10 min to ensure complete paraffin removal and prevent reagent carryover.
  The second xylene bath removes residual paraffin and minimizes carryover from the previous solution.
9 Transfer the slides to xylene III for 10 min using fresh reagent to maximize paraffin dissolution.
  Xylene acts as an organic solvent that removes paraffin, which is not miscible with aqueous solutions. The third xylene bath ensures complete deparaffinization and prepares the tissue for subsequent rehydration steps.
10 Immerse the slides in a xylene/ethanol solution for 10 min to initiate the transition from the organic solvent (xylene) to a polar solvent (ethanol), preventing abrupt solvent incompatibility.
11 Immerse the slides in 100% ethanol to remove residual xylene.
  100% ethanol replaces xylene and begins the rehydration process.
12 Transfer the slides to 90% ethanol to gradually reintroduce water into the tissue.
13 Transfer the slides to 70% ethanol to continue progressive rehydration.
  Graded ethanol solutions prevent osmotic shock and preserve tissue morphology.
14 Rinse the slides in distilled water to complete rehydration and prepare the tissue for aqueous antigen retrieval and immunohistochemical procedures (Bancroft & Stevens, 1990).

#### Antigen retrieval

15 Prepare the 50× EnVision FLEX target retrieval solution, high pH, by diluting 4 ml of the concentrated reagent in 196 ml distilled water to obtain a 1× working solution.
  This alkaline buffer promotes the reversal of formaldehyde‐induced protein cross‐links formed during fixation.
16 Transfer the diluted retrieval solution to a heat‐resistant staining container compatible with pressure heating. Fully immerse the slides in the retrieval solution, ensuring the tissue sections are completely covered and that no air bubbles are trapped on them.
17 Place the container with the slides inside a pressure cooker.
18 Perform heat‐induced epitope retrieval (HIER) by heating under pressure for 8 min.
  High temperature and alkaline pH disrupt methylene bridges formed during formalin fixation, restoring the three‐dimensional conformation of masked antigenic epitopes and improving antibody accessibility.
19 After heating, allow the pressure cooker to cool gradually to room temperature before opening to prevent tissue detachment and sudden temperature shock.
20 Remove the slides carefully and allow them to equilibrate in the retrieval solution for several minutes at room temperature to stabilize antigen exposure.
21 Wash the slides repeatedly with EnVision FLEX wash buffer diluted 1:20 to remove residual retrieval buffer and normalize pH conditions before proceeding to the blocking step.
  This washing step prevents nonspecific background staining caused by residual alkaline buffer.

#### Blocking

22 Prepare a 5% powdered milk blocking solution by dissolving 10 g of powdered milk in 200 ml PBS.
  Powdered milk contains casein and other proteins that occupy nonspecific binding sites, thereby reducing background staining caused by nonspecific antibody interactions.
23 Mix the solution thoroughly until complete dissolution occurs (using a shaker).
24 Incubate all tissue sections, including those designated for negative controls and primary antibody treatment, in the 5% milk solution for 1 hr at room temperature.
  This step blocks nonspecific protein‐binding sites and minimizes background fluorescence.
25 Ensure that the sections remain fully covered with blocking solution during incubation to prevent drying artifacts.
26 Wash the slides carefully with EnVision FLEX wash buffer (1:20 dilution) to remove excess blocking solution while maintaining reduced nonspecific binding conditions.

#### Primary antibody incubation

27 Dilute the biotinylated monoclonal anti‐WASLB antibody 1:50 in BSA.
  BSA stabilizes the antibody and further reduces nonspecific interactions with tissue components.
28 Mix the antibody solution to ensure homogeneity (use vortex for a few seconds).
29 Apply 100 µl of the diluted antibody solution to each slide containing two to three tissue sections, ensuring complete coverage of the sections.
30 Incubate the slides overnight at 4°C in a humidified dark box to prevent evaporation (moisten paper with distilled water and place it inside the dark chamber to maintain humidity).
  Overnight incubation at low temperature enhances specific antigen–antibody binding while reducing nonspecific interactions.
31 Apply the negative control solution to designated slides.
32 Incubate the negative control slides overnight at 4°C under the same conditions as the experimental slides (in a humidified dark box).

#### Secondary detection and counterstaining

33 Wash the tissue sections with EnVision FLEX wash buffer (1:20) to remove unbound primary antibody.
  Thorough washing reduces background fluorescence and improves signal specificity.
34 Incubate the sections for 1 hr at room temperature with streptavidin conjugated to Alexa Fluor 405, ensuring complete coverage of the tissue.
  Streptavidin binds with high affinity to the biotin moiety of the primary antibody, allowing fluorescent visualization of the antigen–antibody complex.
35 Protect the slides from light during incubation to prevent photobleaching of the fluorophore.
36 Wash the slides thoroughly with wash buffer to remove excess conjugate and reduce nonspecific fluorescence.
37 Incubate all sections with DAPI according to the manufacturer's instructions for nuclear counterstaining.
  DAPI binds preferentially to AT‐rich regions of DNA, enabling visualization of cell nuclei and preservation of tissue morphology reference.
38 Rinse the slides gently, if necessary, to remove excess DAPI. This reagent was applied directly onto the tissue sections, and the coverslip was mounted immediately afterward, without requiring incubation or washing steps.
39 Mount coverslips using an appropriate mounting medium compatible with fluorescence.
40 Seal the coverslips with colorless nail polish (store the mounted slides at 4°C in holders wrapped with aluminum foil to prevent light exposure, or inside a dark box). Ideally, wait at least 2 hr for complete drying of the nail polish before observing the slides under the microscope, preventing coverslip displacement.

## COMMENTARY

### Critical Parameters

In this protocol, the time in the pressure cooker, the multiple washing steps, and the antibody and streptavidin dilutions are critical parameters for achieving a successful IHC reaction. In addition, properly preserved tissue and high‐quality histological sections are essential to accurately observe cellular structures and ensure correct staining.

### Troubleshooting

Prior to establishing the final immunohistochemical protocol, multiple experimental parameters were systematically tested and optimized. The following observations may assist other researchers in applying or adapting this protocol to teleost skeletal muscle or related tissues (Table 1).

**Table 1:** Troubleshooting Guide for Immunohistochemistry Reaction

Parameter tested	Conditions evaluated	Outcome	Final decision/recommendation
Heat‐induced antigen retrieval (pressure cooker)	30, 20, 15, and 8 min	Adequate antigen exposure observed only at 8 min (see supplementary document Fig. 1 in Supporting Information)	Use 8 min retrieval; optimal time may vary depending on section thickness and tissue density
Primary antibody dilution	1:100 (commonly reported in literature)	Weak and nonspecific labeling	1:100 not suitable for teleost skeletal muscle; dilution must be empirically optimized
Streptavidin dilution	1:400 (literature‐based)	Insufficient binding to biotinylated primary antibody	Higher concentration required for effective detection (1:200 was the best dilution)
Antibody centrifugation prior to dilution	With vs without centrifugation	No improvement in labeling	Omit centrifugation; homogenize antibody and streptavidin solutions using vortex before application
Sodium borohydride incubation	Aldehyde blocking step tested	Ineffective blocking of cross‐linked aldehyde groups	Exclude from final protocol
Blocking solution composition	Powdered milk in 3% BSA	Inadequate reduction of background staining	Increase powdered milk concentration to 5%
Blocking incubation time	5, 10, 20, 30, and 60 min	60 min produced most consistent reduction of nonspecific binding (see supplementary document Fig. 2 in Supporting Information)	Incubate for 60 min at room temperature
Washing steps	3‐5 min per wash	Essential to remove residual reagents and prevent slide artifacts	Maintain multiple washing steps (3‐5 min each) for optimal specificity

### Understanding Results

In this protocol, WASLB immunolabeling was presented as an example to validate the described method. Accordingly, the staining pattern for each target is expected to vary depending on the cellular localization of the protein under investigation. Users should therefore interpret staining results within the context of the known or predicted cellular or subcellular localization of the protein of interest or describe them as novel findings if no prior reports of its localization are available in the literature.

**Figure 2:**
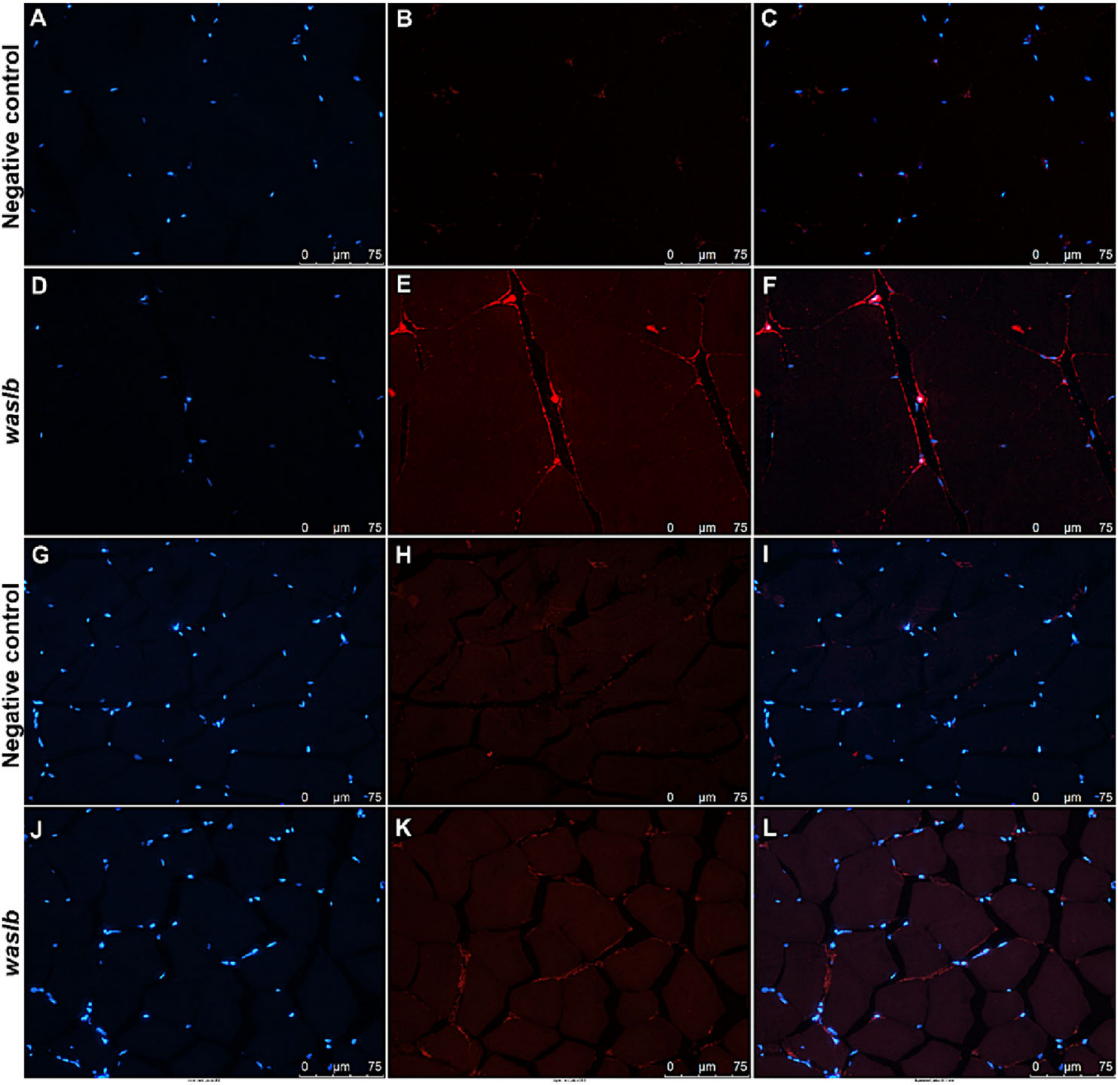
Immunohistochemical detection of WASLB in skeletal muscle of pacu (*Piaractus mesopotamicus*) (**A**‐**F**) and zebrafish (*Danio rerio*) (**G**‐**L**) using a species‐specific biotinylated primary antibody against WASLB. (**A**), (**D**), (**G**), and (**J**) show nuclear counterstaining with DAPI. (**B**), (**E**), (**H**), and (**A**) show WASLB immunolabeling revealed by fluorophore‐conjugated streptavidin. (**C**), (**F**), (**I**), and (**L**) show merged images of DAPI and streptavidin labeling. (**A**‐**C**) and (**G**‐**I**) represent negative controls, in which sections were incubated with the negative control solution only.

For WASLB, the protocol was successfully implemented, and specific labeling was characterized by a continuous, non‐punctate signal outlining the sarcolemma of skeletal muscle fibers (five biological replicates, with three sections per histological slide) in both pacu (*n* = 5) and zebrafish (*n* = 5). Labeling was also observed in putative activated satellite cells located within the endomysium or perimysium, the connective tissue surrounding muscle fibers. Minimal cytoplasmic background was detected, while DAPI nuclear counterstaining clearly revealed multiple intact myonuclei positioned at the fiber periphery without interfering with antigen detection. In negative controls, in which primary antibody binding sites were blocked with the immunizing peptide, no specific red signal was detected. The presence of staining in control sections would indicate nonspecific binding, insufficient blocking, interference from endogenous biotin, or excessive antibody concentration. It is important to note that the peptide‐to‐antibody ratio required for negative blocking may vary depending on the characteristics of the synthesized antibody. Representative examples of antibody labeling and the corresponding negative controls for each species are shown in Figure 2.

Based on these results, an immunohistochemical protocol optimized for the skeletal muscle of two teleost species was established and validated using a biotinylated monoclonal antibody common to both species and specific for WASLB. Although direct detection systems are generally considered less sensitive than indirect methods employing secondary antibodies, each approach offers advantages depending on experimental objectives and technical requirements (Im et al., 2019). In the present study, direct biotin–streptavidin detection eliminated the need for a secondary antibody, thereby reducing potential cross‐reactivity and avoiding additional optimization steps beyond those required for the primary antibody. These considerations are particularly relevant when performing immunohistochemistry in non‐mammalian species (Ramos‐Vara, 2005; Taylor & Levenson, 2006). Moreover, endogenous biotin interference has been reported predominantly in metabolically active tissues, such as liver and kidney, with limited evidence in skeletal muscle, particularly in paraffin‐embedded samples (Kim et al., 2002; Miller et al., 1997), further supporting the suitability of this detection strategy for the present protocol. The absence of nonspecific labeling in negative controls further confirms the appropriateness of this approach for teleost skeletal muscle tissue.

Importantly, a rigorous peptide competition assay provided robust validation of antibody specificity in negative controls. Although this methodological step remains underreported in fish immunohistochemistry studies, it is widely recommended to ensure antibody reliability (Bordeaux et al., 2010; Saper, 2009) and is particularly relevant given interspecific differences in fixation sensitivity and epitope preservation (Bergh, 2016; Ramos‐Vara, 2005; Shi et al., 2007). From a methodological standpoint, extensive optimization was required to establish effective antigen retrieval conditions, antibody dilution, blocking strategies, and washing steps, as routinely performed in conventional immunohistochemical assays, rendering this protocol reproducible. Variations in signal intensity may occur depending on fixation time, antigen retrieval efficiency, or antibody dilution. However, once optimal conditions are established, the localization pattern of the target protein should remain consistent across sections and biological replicates. A weak but clearly localized signal is generally preferable to intense staining accompanied by background (Bordeaux et al., 2010). Overstaining, patchy labeling, or uniform fluorescence throughout the section typically indicate suboptimal washing, inadequate blocking, or excessive primary antibody concentration (Ramos‐Vara, 2005). Conversely, absence of signal in experimental samples may be associated with ineffective antigen retrieval, over‐fixation or fixative type, epitope masking, or excessive antibody dilution. In this context, reproducibility of the protocol was confirmed by consistent localization, low background, and absence of signal in negative controls, which constitute the primary criteria for validating successful implementation of the method (Taylor & Levenson, 2006). Furthermore, future optimization and application of western blotting in this experimental model could provide complementary evidence to support the findings obtained through standardized immunohistochemistry. The lack of protein‐level validation by western blotting represents a limitation of the present study and highlights an important avenue for future research.

Taken together, the methodological parameters described herein resulted in a reproducible protocol for the immunohistochemical analysis of teleost skeletal muscle. These conditions may serve as a practical guide for application to additional molecular targets and other fish species.

### Time Considerations

To carry out this protocol, 1 day is required for slide preparation and control setup, 1 day for the IHC reaction, and 1 additional day for detection and slide mounting.

### Conclusion

The protocol described herein provides a reliable and reproducible strategy for the immunohistochemical detection of WASLB protein in teleost skeletal muscle. By integrating a custom anti‐WASLB antibody developed from conserved teleost sequences with optimized antigen retrieval procedures and specificity controls, this methodology enables accurate and consistent protein localization in both pacu and zebrafish tissues.

Although further validation is warranted for additional targets and species, the present work establishes a valuable methodological foundation for protein‐level investigations in teleosts and other non‐mammalian vertebrates. Given the scarcity of standardized immunohistochemical protocols for these groups, this approach represents a significant contribution to the field and may facilitate future advances in comparative physiology, developmental biology, and muscle research.

### Author Contributions

**Érika Stefani Perez**: Conceptualization; data curation; formal analysis; funding acquisition; investigation; methodology; project administration; resources; validation; visualization; writing—original draft; writing—review and editing. **Matheus Naia Fioretto**: Formal analysis; methodology; validation; visualization. **Bruna Tereza Thomazini Zanella**: Visualization. **Rafael Henrique Nóbrega**: Resources; visualization. **Bruno Oliveira da Silva Duran**: Visualization. **Maeli Dal Pai‐Silva**: Conceptualization; data curation; funding acquisition; methodology; project administration; resources; supervision; visualization; writing—review and editing.

### Conflict of Interest

The authors declare no potential conflicts of interest with respect to the research, authorship, and/or publication of this article.

## Supporting information


*The supplementary material document includes calculations regarding the excess of peptide relative to the antibody required for the blocking solution. In addition, it provides important information about unsuccessful attempts that may be relevant to users*.

## Data Availability

Data available in a supplementary material document (see Supporting Information).
