# Supplementary material for: Standardization of Immunohistochemistry Using an Anti‐WASLB Antibody in the Skeletal Muscle of Teleost Fish
Source: Curr Protoc. 2026 Jun 16;6(6):e70404. doi: 10.1002/cpz1.70404 (PMC13270420; doi:10.1002/cpz1.70404)
Supplement: Supplementary file 1 — The supplementary material document includes calculations regarding the excess of peptide relative to the antibody required for the blocking solution. In addition, it provides important information about unsuccessful attempts that may be relevant to users. [file CPZ1-6-0-s001.docx]

**Supplementary Material**

**Calculation of Molar Excess for the Negative Control Solution**

Considering these concentrations, using 1 µL of antibody for a 20,000:1 molar ratio (peptide:antibody):

*1. The first step is to calculate the antibody molar amount:*

(1 × 10⁻⁶ g) / 145,000 g/mol = 6.9 × 10⁻¹² mol = 6.9 pmol

*2. Then, it is necessary to calculate the peptide molar amount:*

(308.8 × 10⁻⁶ g) / 2,239.5 g/mol = 1.38 × 10⁻⁷ mol = 138 nmol

*3. After calculating the molar amount of both, we calculate the molar ratio:*

MR = 138,000 pmol / 6.9 pmol = 20,000

In other words, using 1 µg of antibody and 308.8 µg of peptide, a 20,000-fold molar excess of peptide relative to the antibody is achieved.

*4. Thus, for a 20,000× molar excess of peptide:*

6.9 pmol × 20,000 = 138,000 pmol = 138 nmol

138 nmol × 2,239.5 g/mol = 3.09 × 10⁻⁴ g = 309 µg

**Optimization steps and troubleshooting for teleost skeletal muscle IHC**

Prior to establishing the final immunohistochemical protocol, multiple experimental parameters were systematically tested and optimized. The following observations may assist other researchers in applying or adapting this protocol to teleost skeletal muscle or related tissues:

1. Heat-induced antigen retrieval in a pressure cooker was tested for 30, 20, 15 and 8 minutes. Effective antigen exposure was achieved only with an 8-minute retrieval period (Supplementary Figure.1). It should be noted that optimal retrieval time may vary depending on section thickness and tissue density.

2. The antibody dilution commonly reported in the literature (1:100) resulted in weak and nonspecific labeling and was therefore unsuitable for detection of the target protein in teleost skeletal muscle.

3. Streptavidin dilution at 1:400, as described in previous studies, was insufficient for effective binding to the biotinylated primary antibody.

4. Centrifugation of the antibody prior to dilution did not improve tissue labeling. Consequently, antibody and streptavidin solutions were homogenized using a vortex mixer before application.

5. Incubation with sodium borohydride solution did not effectively block cross-linked aldehyde groups and was excluded from the final protocol.

6. Blocking with powdered milk diluted in 3% BSA was ineffective in reducing background staining, requiring an increased concentration of 5% powdered milk.

7. Blocking incubation with powdered milk in times of 5, 10, 20, 30, and 60 minutes were evaluated. Incubation for 60 minutes at room temperature yielded the most consistent reduction of nonspecific binding (Supplementary Figure.2).

8. Multiple washing steps (3–5 minutes each) were essential for removing residual reagents and minimizing slide artifacts, resulting in clean and specific immunolabeling.


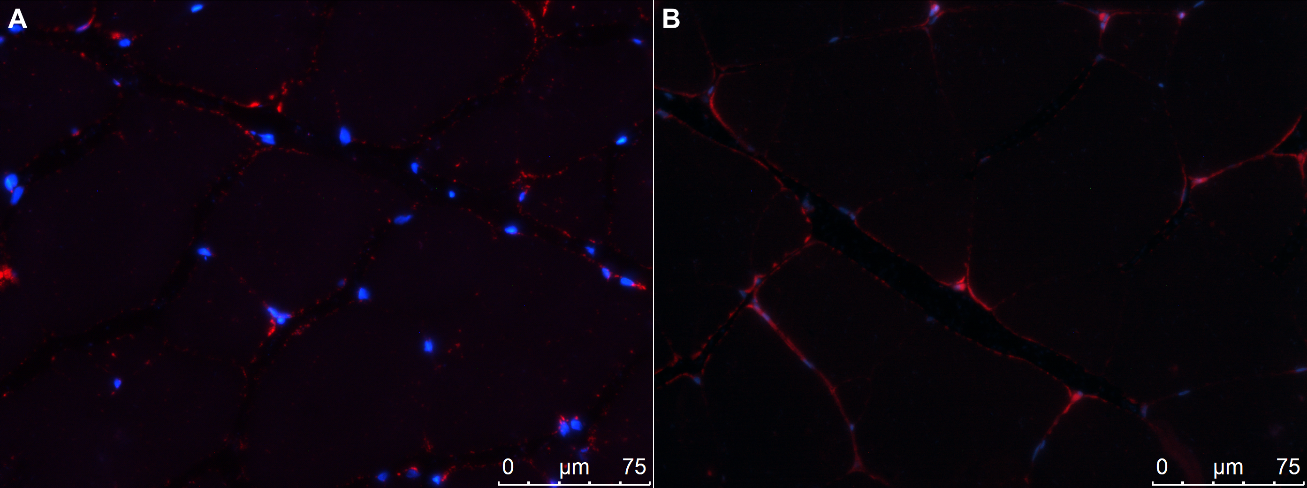


**Supplementary Figure 1.** Immunohistochemical detection of WASLB in fish skeletal muscle. (A) Representative sections showing punctate immunolabeling of the WASLB antibody along the sarcolemma (muscle fiber membranes) following heat-induced antigen retrieval performed in a pressure cooker for 30, 20, and 15 minutes. (B)Immunohistochemical labeling of WASLB after heat-induced antigen retrieval in a pressure cooker for 8 minutes.


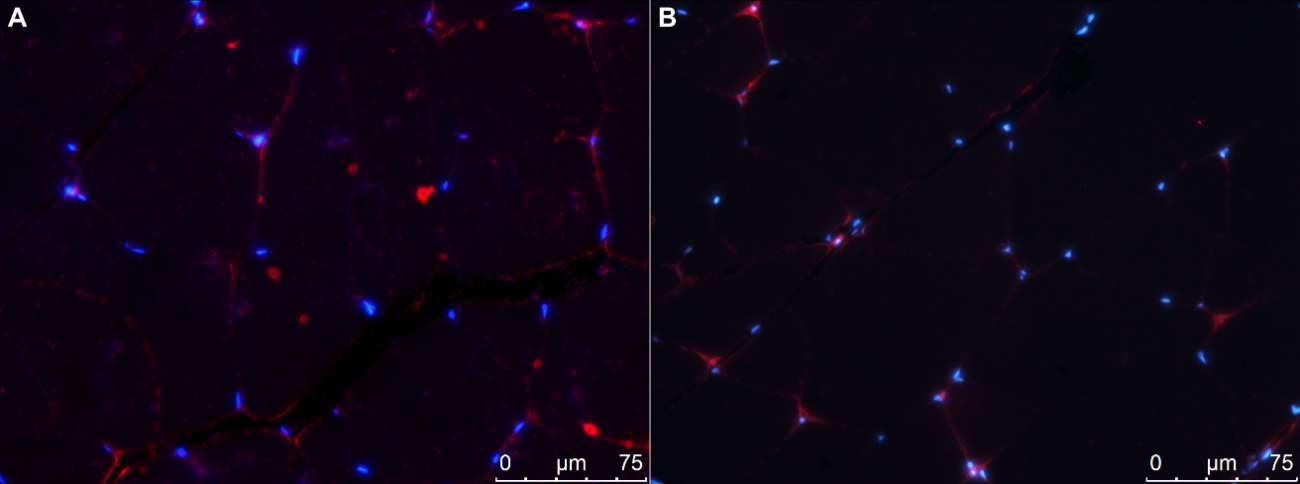


**Supplementary Figure. 2** Immunohistochemical detection of WASLB in fish skeletal muscle. (A) Representative histological sections incubated with powdered milk blocking solution for less than 60 minutes, showing nonspecific labeling and background signal. (B) Sections incubated with powdered milk blocking solution for 60 minutes, showing reduced background and improved specificity of WASLB immunolabeling.
